# Immunohistochemical Profile for Unknown Primary Adenocarcinoma

**DOI:** 10.1371/journal.pone.0031181

**Published:** 2012-01-27

**Authors:** Kenji Hashimoto, Yuko Sasajima, Masashi Ando, Kan Yonemori, Akihiro Hirakawa, Koh Furuta, Hitoshi Tsuda, Yasuhiro Fujiwara

**Affiliations:** 1 Department of Breast Oncology and Medical Oncology, National Cancer Center Hospital, Tokyo, Japan; 2 Department of Pathology and Clinical Laboratories, National Cancer Center Hospital, Tokyo, Japan; 3 Department of Management Science, Graduate School of Engineering, Tokyo University of Science, Tokyo, Japan; Health Canada, Canada

## Abstract

**Background:**

Development of tailored treatment based on immunohistochemical profiles (IPs) of tumors for cancers of unknown primary is needed.

**Methodology/Principal Findings:**

We developed an algorithm based on primary known adenocarcinoma for testing sensitivity and specificity. Formalin-fixed paraffin-embedded tissue samples from 71 patients of unfavorable subsets of unknown primary adenocarcinoma were obtained. We examined 15 molecular markers using the algorithm incorporating these IPs and classified the tumours into 9 subsets based on the primary tumour site. The sensitivity and specificity of this algorithm were 80.3% and 97.6%, respectively. Apparent primary sites were lung in 17 patients, digestive organs in 13, gynecological organs in 9, prostate in 7, liver or kidney in 6, breast in 4, urothelial organ in 2, biliary tract and pancreatic profile in none, and unclassified in 13. The response rate to chemotherapy was highest for the gynecological IPs. Patients with gynecological or lung cancer IPs had longer median progression-free survival than those with others: 11.2 months for gynecological IPs (p<0.001) and 6.8 months for lung IPs (p = 0.05). Lung, digestive, prostate, and gynecological profiles were associated with significantly longer median survival time than the other profiles. Multivariate analysis confirmed that the IPs were independent prognostic factors for survival.

**Conclusions/Significance:**

The IPs identified in this study can be used to further stratify patient prognosis for unfavorable subsets of unknown primary adenocarcinoma.

## Introduction

Cancers of unknown primary site (CUPs) account for approximately 3% of all malignant neoplasms [1]. CUPs are defined as a heterogeneous group with metastatic disease for which the site of origin cannot be identified at the time of diagnosis despite careful clinical and laboratory examination [2]. Adenocarcinoma accounts for about 50% of CUPs, and unfavorable subtypes of heterogeneous adenocarcinomas are generally treated with platinum doublet chemotherapy regimens, with 6–12 months median overall survival [3], [4], [5], [6].

Chemotherapy regimens vary by institute for unfavorable subtypes of CUPs; however, the prognosis remains poor. Because CUPs comprise heterogeneous neoplasms, investigators have focused on developing tailored treatments using modern approaches, identifying molecular targets for CUP-specific therapy, identifying the primary cancer site and applying disease-oriented therapy, or identifying the primary site by gene profiling assay using complementary deoxyribonucleic acid (cDNA) or oligonucleotide microarrays [7], [8]. An alternative approach is staining samples using immunohistochemistry (IHC) to determine the primary tumor site. Recent studies have demonstrated that IHC can identify unique subsets of CUPs, and that organ-specific chemotherapy for these subsets may have benefit [9]. However, classification of CUPs by IHC using conventional antibodies requires further development to improve sensitivity and specificity in determining the primary site [10].

Through recent advancements in IHC, additional organ-specific antibodies have become available [9], [11], [12], [13], [14], [15], [16], [17], [18], [19], [20], [21], [22], [23], [24], including the estrogen receptor (ER), progesterone receptor (PgR), mammaglobin, gross cystic disease fluid protein-15 (GCDFP-15), CDX2, thyroid transcription factor-1 (TTF-1), Wilms tumor susceptibility gene 1 (WT-1), paired box gene 8 (PAX8), prostate-specific antigen (PSA), and uroplakin with conventional antibodies including cytokeratin (CK) 7 and CK20. Use of these biomarkers has the potential to identify the primary tumor site with greater sensitivity and specificity [25], [26]. Furthermore, K-*ras* gene mutation analysis may assist in determining the primary site when combined with IHC for pancreatic or bile duct cancer [27], [28]. Improving treatment of CUPs requires identification of the primary tumor site using molecular markers and application of primary tumor site-specific treatment. We selected adenocarcinomas of unknown primary site for study and conducted the present single-center retrospective biomarker analysis to provide the basis for an upcoming prospective clinical trial.

## Results

### Patient characteristics and immunohistochemistry profile

Patient characteristics are listed in Table 1. Nearly 50% of biopsy samples were taken from lymph nodes. Other biopsy sites included lacrimal grand, chest wall, vaginal, and brain. A carboplatin and paclitaxel regimen was used most frequently (40%), followed by a carboplatin and irinotecan regimen (31%). The carboplatin and irinotecan, carboplatin and S1, and cisplatin and docetaxel regimens were prospectively evaluated in our phase II trials [5], [6] or ongoing prospective trial.

**Table 1:** Patient characteristics at diagnosis.

Variable		*n* (%)
Age	Median (range)	62 (36–78)
	≥65 yr	25 (35)
	<65 yr	46 (65)
Gender	Male	40 (56)
	Female	31 (44)
Performance status	0	3 (4)
	1	39 (55)
	2	29 (41)
Sites of biopsy	Lymphnodes	38 (54)
	Bone or Bone marrow	10 (14)
	Liver	6 (9)
	Gastrointestinal tract	4 (6)
	Skin	2 (3)
	Adrenal gland	2 (3)
	Others	8 (10)
Bone metastasis	Yes	13 (18)
	No	58 (82)
Liver metastasis	Yes	6 (9)
	No	65 (91)
Lung metastasis	Yes	12 (17)
	No	59 (83)
Lymph node metastasis	No	18 (25)
	Yes	53 (75)
Treatment regimen	Cisplatin or carboplatin/docetaxel	16 (22)
	Carboplatin/irinotecan	22 (31)
	Carboplatin/paclitaxel	29 (41)
	Carboplatin/S1 or FOLFOX	4 (6)

FOLFOX: 5FU, oxaliplatin, and *l*-leukovorin.

**Figure 1:**
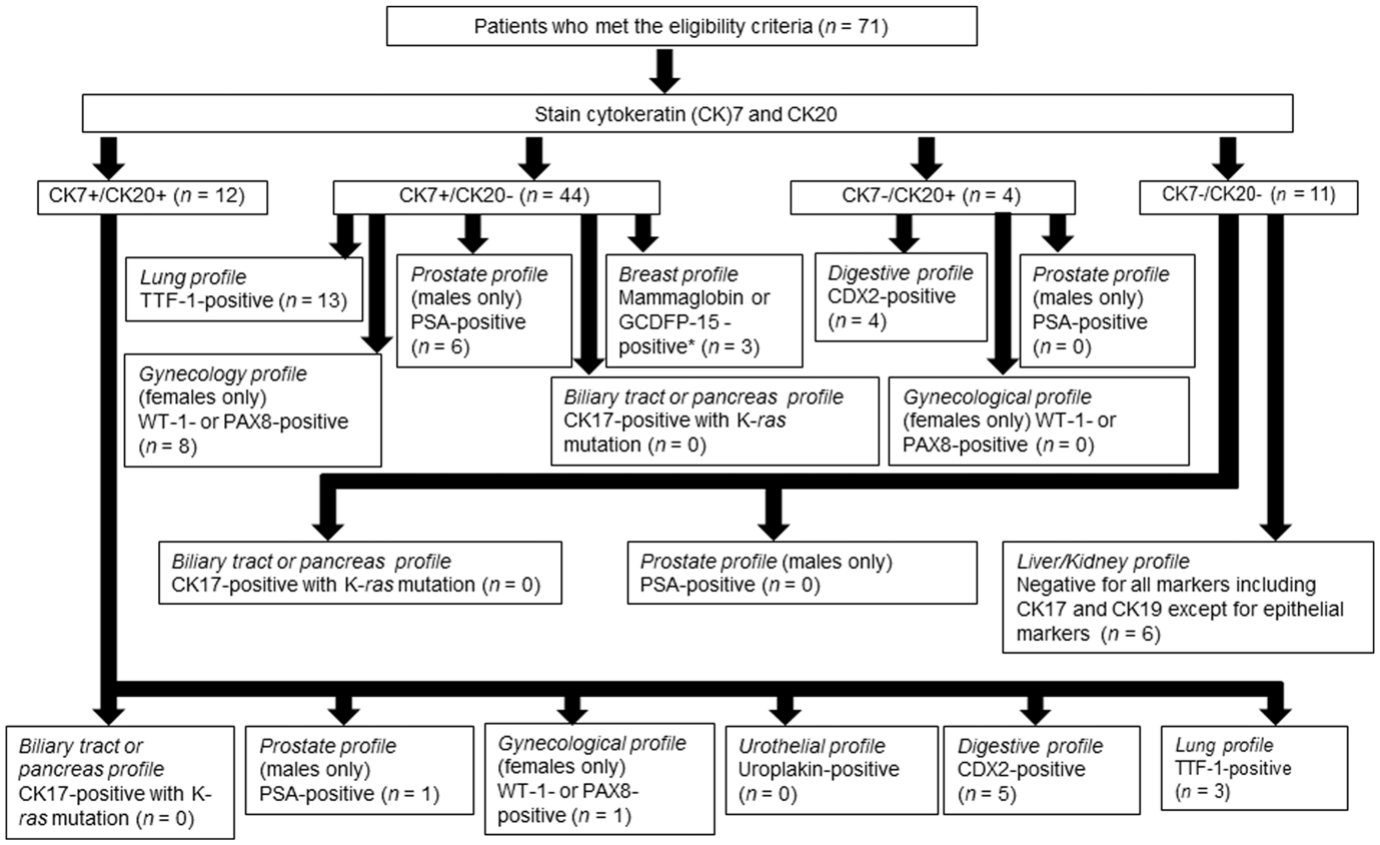
Identification of the primary tumor site by immunohistochemistry and gene analysis. Thirteen patients were not sorted to specific profiles and an additional 8 patients were not classified into the profiles in the manner defined by the algorithm. Two patients presenting with both CK7− and CK20-positive were classified into an unclassified profile. Ten patients with CK7-positive and CK20-negative were classified into an unclassified profile. Three patients with CK7-positive and CK20-negative were classified into the digestive (*n* = 1) and urothelial (*n* = 2) profiles. One patient with both CK7− and CK20-negative was classified into the unclassified profile. Four patients with both CK7− and CK20-negative were classified into the digestive (*n* = 3) and lung (*n* = 1) profiles. Footnote: * Estrogen receptor- or progesterone receptor-positive with CK19-positive.

We examined the sensitivity and specificity of the algorithm (Figure 1) by using known primary tumors which were surgically resected; 10 lung adenocarcinoma; 11 ovarian adenocarcinoma; 10 endometrial adenocarcinoma; 10 breast adenocarcinoma; 10 prostate adenocarcinoma; 10 urothelial adenocarcinoma; 10 colorectal adenocarcinoma; 10 gastric adenocarcinoma; 11 pancreatic adenocarcinoma; 5 biliary tract adenocarcinoma; 10 hepatocellular adenocarcinoma; and 10 renal cell adenocarcinoma. Overall sensitivity and specificity of this algorithm to identify the primary site were 80.3% and 97.6%, respectively. For each subset, the sensitivity and the specificity were as follows; lung profile, 100% and 100%; gynecology profile, 100% and 95.9%; breast profile, 90% and 100%; prostate profile, 100% and 94.5%; urothelial profile, 20% and 99%; digestive profile, 85.7% and 96.9%; biliary tract or pancreas profile, 0 and 94.7%; and liver or kidney profile, 80% and 100%, respectively.

**Figure 2:**
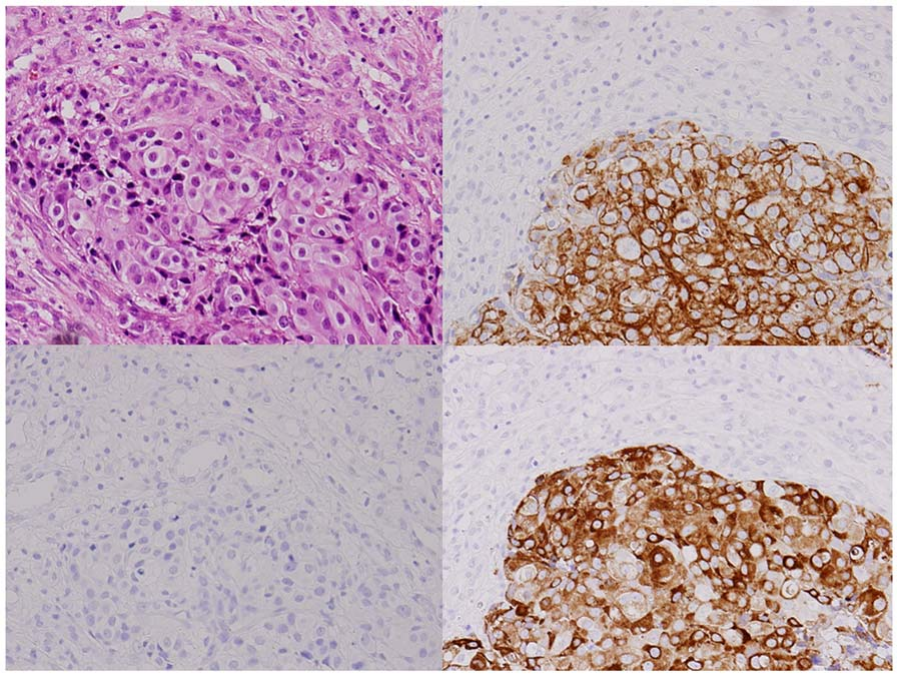
An adenocarcinoma showing typical presentation of the breast profile by immunohistochemistry. Hematoxylin-eosin stain (left upper), cytokeratin (CK)7 (right upper), CK20 (left lower), and mammaglobin (right lower). CK7 and mammaglobin are positive in the cytoplasm of tumor cells (original magnification ×200).

**Figure 3:**
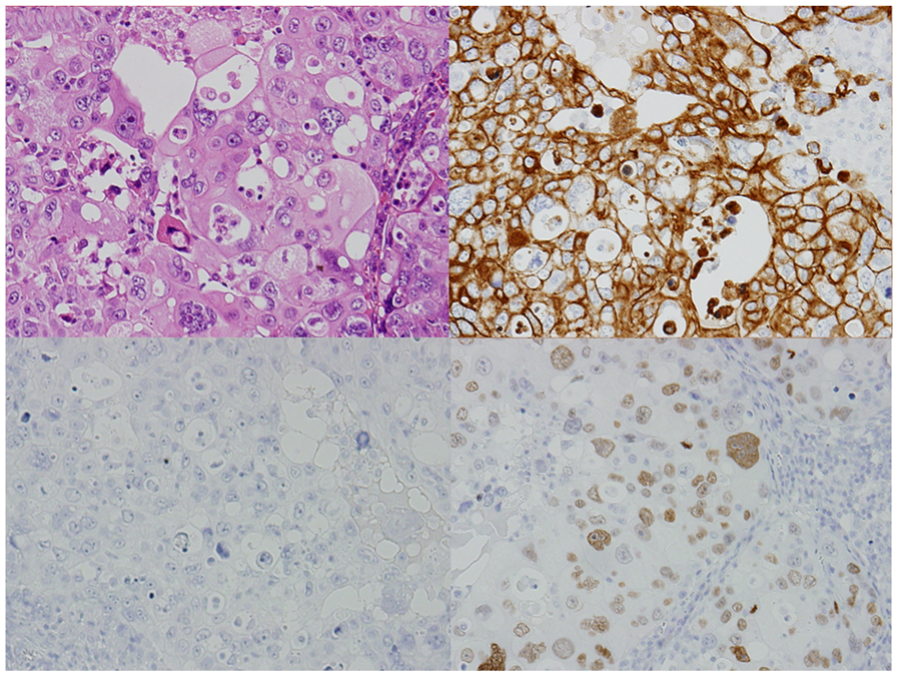
An adenocarcinoma showing typical presentation of the lung profile by immunohistochemistry. Hematoxylin-eosin stain (left upper), cytokeratin (CK)7 (right upper), CK20 (left lower), and thyroid transcription factor-1 (TTF-1) (right lower). CK7 and TTF-1 are positive in the cytoplasm and nuclei of tumor cells, respectively (original magnification ×200).

The primary tumor sites based on the immunohistochemical profiles (IPs) for the 71 unknown primary patients were the lung for 17 patients, digestive organs for 13, gynecological organs for 9, prostate for 7, liver/kidney for 6, breast for 4, urothelial for 2, and were not unclassified for 13 patients (Table 2).Figures 2 and 3 show typical IHC results for the breast and lung profiles.

**Table 2:** Primary tumor site of unfavorable subsets of adenocarcinoma of unknown primary determined by a immunohistochemistry profile of 15 markers and the relationship to response rate to platinum doublet regimens.

Profile	*n* (%)	Complete response	Partial response	Response rate (%)
Lung	17 (24)	2	3	17.6
Digestive	13 (18)	0	2	15.4
Gynecological	9 (13)	3	3	66.7
Prostate	7 (10)	0	2	28.6
Liver/Kidney	6 (8)	0	2	33.3
Breast	4 (6)	0	1	25.0
Urothelial	2 (3)	0	0	0
Not identified	13 (18)	2	2	30.8

The CK7-positive and CK20-negative cohort was the most frequent (Figure 1). We found that 7 tumors did not exhibit profiles in accordance with the algorithm: four were CDX2-positive but CK20-negative and were classified into the digestive profile, one was negative for both mammaglobin and GCDFP-15 and all other markers other than CK19 and ER (Allred score was 7) [29] and was classified into the breast profile, and two were positive for uroplakin and both negative for CK7 and CK20 and were classified into the urothelial profile. CK17 was also positive in 9 patients according to the algorithm that did not have K-*ras* gene mutations. For remaining 13 patients, a specific primary profile was not identified. Among the 17 lung profile patients, 15 samples were available for *EGFR* mutation analysis and one demonstrated mutation of codon 858 in exon 21. For digestive IP, 9 samples were available for K-*ras* mutation analysis and 3 of these were positive (codon12 TGT, codon12GTT, and codon12 GAT).

### Response evaluation and survival analysis

**Figure 4:**
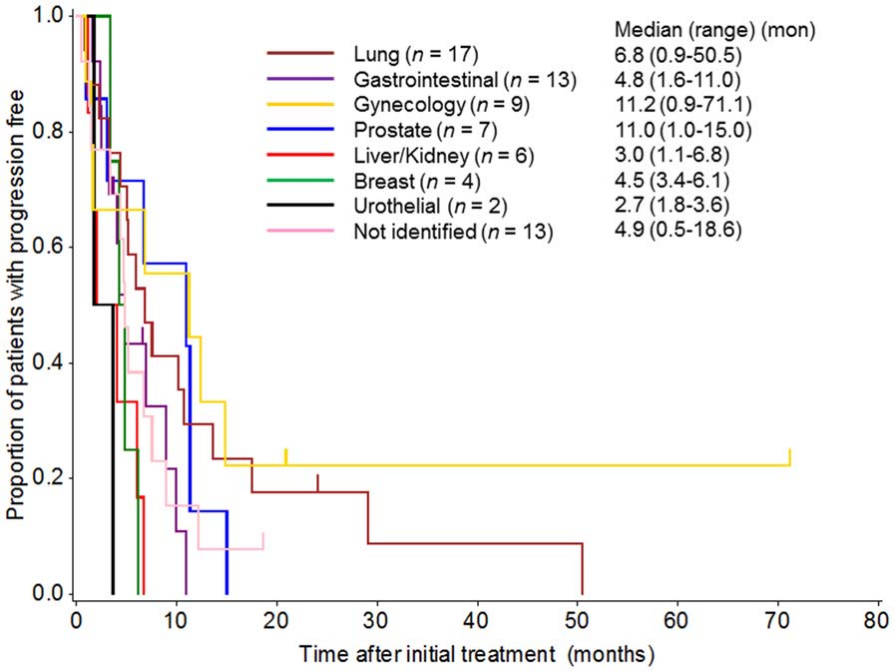
Progression-free survival curve by the Kaplan-Meier method for the groups with each primary site as classified by the immunohistochemistry profiles.

**Figure 5:**
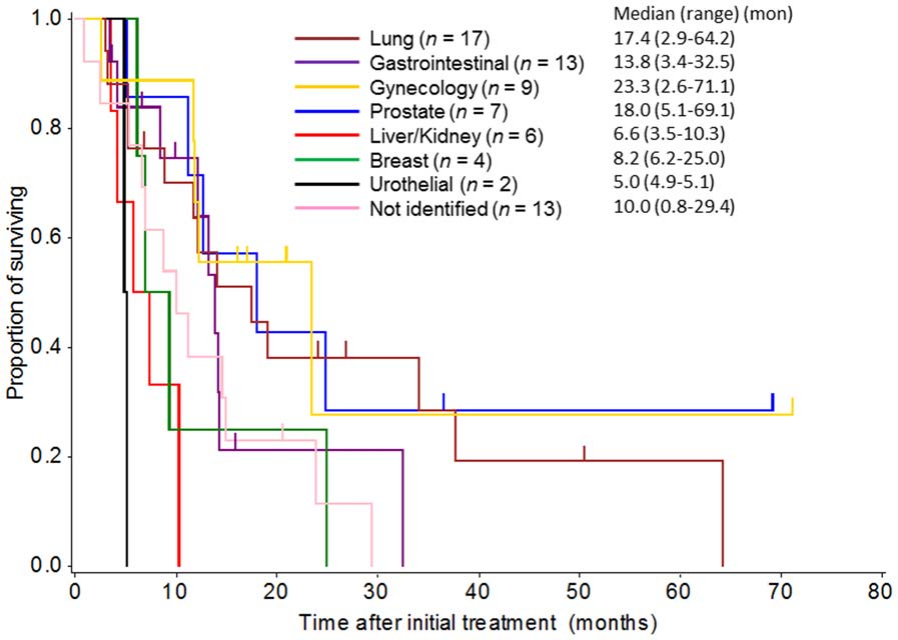
Overall survival curve by the Kaplan-Meier method for the groups with each primary site as classified by the immunohistochemistry profiles.

The overall response rate was 31%. Response rates by profile are listed in Table 2. A higher response rate was observed for the gynecological profile (67%) than for the other profiles. Progression-free survival varied significantly by IP. The median PFS was 6.8, 11.2, and 11.0 months for the lung, gynecological, and prostate IPs, respectively, while those of the other IPs, including the digestive, liver/kidney, breast, urothelial, prostate, and unclassified profiles were 4.8, 3.0, 4.5, 2.7, and 4.9 months, respectively (Figure 4). Univariate and multivariate analyses of PFS are shown in Table 3. In the multivariate analysis, PFS was significantly longer for the gynecological and lung profiles than for the other profiles. Likewise, the median survival time was 23.3, 17.4, 18.0, and 13.8 for the gynecological, lung, prostate, and digestive IPs, respectively, while others including the liver/kidney, breast, urothelial, and unclassified IPs were 6.6, 8.2, 5.0, and 10.0 months, respectively (Figure 5). In the multivariate analysis, bone metastasis, poor performance status, and male patients, in addition to the IP, were independent prognostic factors. Lung, gynecological, digestive, and prostate profiles had significantly longer survival times than the other profiles (Table 4).

**Table 3:** Prognostic significance for progression-free survival of the immunohistochemistry profile and other parameters by Cox univariate and multivariate analyses.

		Univariate			Multivariate		
		HR	95%CI	*p*	HR	95%CI	*p*
No. of	1–2 organs						
metastases	≥3 organs	1.12	0.66–1.90	0.67	0.92	0.43–1.99	0.84
Liver	No						
metastasis	Yes	1.30	0.55–3.04	0.55	1.36	0.51–3.62	0.54
Bone	No						
metastasis	Yes	1.80	0.94–3.45	0.08	1.73	0.77–3.89	0.19
Performance	0–1						
score	2	2.86	1.71–4.77	<0.001	3.32	1.81–6.08	<0.001
Gender	Female						
	Male	0.70	0.43–1.14	0.15	0.33	0.17–0.66	0.002
Age	≥65						
	<65	0.62	0.37–1.10	0.08	0.57	0.32–1.03	0.06
Profile	Liver/Kidney						
	Prostate	0.30	0.10–0.93	0.04	0.38	0.09–1.52	0.17
	Digestive	0.52	0.19–1.42	0.20	0.44	0.13–1.46	0.18
	Breast	0.78	0.22–2.77	0.70	0.48	0.13–1.82	0.28
	Urothelial	1.58	0.31–7.98	0.58	0.46	0.08–2.58	0.38
	Lung	0.27	0.10–0.72	0.009	0.31	0.10–1.00	0.05
	Gynecological	0.18	0.06–0.59	0.004	0.08	0.02–0.30	<0.001
	Not identified	0.44	0.16–1.20	0.11	0.40	0.14–1.21	0.11

HR, hazard ratio; CI, confidence interval; PS, performance status.

**Table 4:** Prognostic significance for survival of the immunohistochemistry profile and other parameters by Cox univariate and multivariate analyses.

		Univariate			Multivariate		
		HR	95%CI	*p*	HR	95%CI	*p*
No. of metastasis	1–2						
	≥3	1.42	0.82–2.45	0.21	1.01	0.44–2.29	0.99
Liver metastasis	No						
	Yes	1.25	0.53–2.96	0.61	1.11	0.37–3.30	0.86
Bone metastasis	No						
	Yes	2.08	1.03–4.19	0.04	3.52	1.44–8.64	0.01
PS	0–1						
	2	1.89	1.11–3.21	0.02	1.99	1.05–3.78	0.04
Gender	Female						
	Male	0.64	0.38–1.09	0.10	0.46	0.23–0.95	0.04
Age	≥65						
	<65	0.54	0.3–0.97	0.04	0.56	0.28–1.12	0.1
Profile	Liver/Kidney						
	Prostate	0.14	0.04–0.48	0.002	0.09	0.02–0.45	0.003
	Digestive	0.27	0.09–0.81	0.02	0.23	0.06–0.81	0.02
	Breast	0.41	0.11–1.50	0.18	0.31	0.07–1.28	0.11
	Urothelial	2.24	0.42–11.9	0.34	1.50	0.23–9.58	0.67
	Lung	0.18	0.06–0.50	0.001	0.20	0.05–0.72	0.01
	Gynecological	0.13	0.04–0.46	0.001	0.09	0.02–−0.35	0.001
	Not identified	0.36	0.13–1.01	0.05	0.37	0.12–1.09	0.07

HR, hazard ratio; CI, confidence interval; PS, performance status.

## Discussion

In this study, we developed a panel for identifying the primary cancer origin of CUPs using our stocked tissue samples and through immunohistochemical profiling along with gene mutation analysis. The sensitivity and specificity of the panel validated by primary known adenocarcinomas were 80.3% and 97.3%. Using this panel, 81.7% of the patients were classified as having a specific primary tumor profile. We then analyzed clinical outcomes according to the panel. The response rate was higher for patients with the gynecological profile than for the other patients. The PFS was also significantly longer for patients with the gynecological and lung profiles than for those with the liver/kidney profile. The multivariate analysis revealed that patients with the gynecological, lung, prostate, and digestive profiles had significantly longer survival than the other patients. Clinical course of survival seems to be consistent with diagnosis of their primary cancers.

In this analysis, we used organ-specific antibodies, including TTF-1 for lung cancer, WT-1 or PAX8 for gynecological cancers, mammaglobin or GCDFP-15 for breast cancer, PSA for prostate cancer, CDX2 for gastrointestinal cancers, and uroplakin for urothelial cancers. In validation of the panel by using these markers, lung, gynecology, breast, prostate, digestive, and liver/kidney profile had high sensitivity and specificity. Notably, liver/kidney profile which organ specific markers are currently unavailable achieved high sensitivity and specificity by deleting other possibilities with CK7 and CK20 negativity. However, urothelial and biliary tract or pancreatic profile had lower sensitivity compared to others. For these profiles, alternative approaches may have some value [7], [8].

These antibodies used in this analysis are valuable for the cohort with adenocarcinoma, because the majority of other cancers that arise from the head and neck, esophagus, and uterine cervix can be excluded. We did not divide the liver/kidney profile into liver and kidney groups, as the first-line treatment for these metastatic diseases is similar [30], [31]. Further, we consider gynecologic profile may not be necessary to be classified into ovary, endometrial, and cervical adenocarcinoma in the situation of adenocarcinoma of unknown primary because chemotherapies for these cancer become similar in advanced disease [32], [33], [34]. The possibilities of this story may be applied to pancreatobiliary tract cancer and digestive cancer when limited to adenocarcinoma of unknown primary [35], [36], [37], [38].

### The algorithm we generated for orienting primary has value

Immunohistochemistory is generally done in routine work for the diagnosis of adenocarcinoma of unknown primary in many cancer centers. Therefore, there is no additional skill or tool in the procedure of diagnosis [10], [25]. Previously, Dannis *et al.* developed algorithm to identify the useful antibodies, specific for primary sites [25]. They developed diagnostic panel to examine 7 primary site (breast, colon, lung, ovary, pancreas, prostate, and stomach) by using FFPE samples of primary known cancers, but liver and kidney origin cancer were not included. The accuracy of diagnosis is 88%, however, the problem is that the algorithm is not applied for unknown primary cancer and the clinical outcomes divided by the algorithm is unknown. Therefore, the beneficiary to apply the algorithm for identification of unknown primary cancer is unclear. Centeno *et al.* also did the similar approach to Dannis by using origin known cancers [39]. They also excluded hepatocellular carcinoma and renal cell carcinoma. The outcome of the algorithm is not validated to unknown primary cancer in relation to clinical outcomes. We developed our panel referencing to their result in part and specified to adenocarcinoma of unknown primary including hepatocellular carcinoma and renal cell carcinoma, and the results of the algorithm for unknown primary adenocarcinoma were consistent with the clinical outcomes of primary known cancers.

Horlings *et al.* reported identification of primary site of unknown primary adenocarcinoma by oligonucleotide microarray [8]. The accuracy was 83%, however, lung, clear cell ovary, pancreas, and stomach origin were misidentified up to 100%. Interestingly, they used immunohistochemical result of unknown primary to orient primary as a reference. However, the immunohistochemical reference arm they used was poor as only a few markers were stained. They also presented a single case study according to gene expression profile but the clinical outcomes for others are unclear. Varadhachary *et al.* used 10 gene markers by real time PCR to identify the primary site in cancer of unknown primary [40]. They did not show the validated result of the panel in known primary cancer. RNA was lost in 13% before testing and the yield of orientation was somewhat low (approximately 70%). They also used immunohistochemistry as a reference including CDX2 for colon cancer, TTF-1 for lung cancer. For ovarian cancer identified by their panel, survival seems not consistent with clinical features of ovarian cancer.

This study has some limitations. First, the prognostic value of each IP was potentially underpowered, as the number of patients in each subgroup was somewhat small, not allowing the response rate, PFS, and OS to be compared to historical control data. Second, the results need to be validated in a prospective manner by applying standard treatments for identified primary profiles, to go beyond simply identifying prognostic factors for unknown primary adenocarcinoma. Further biomarker investigation may be valuable for subgroups other than the *EGFR* mutation for the lung profile, the K-*ras* mutation for the digestive profile, and human epidermal growth factor receptor-2 **overexpression for breast profile**.

In this study, we revealed the prognostic value of a panel composed of immunohistochemistry profiles for patients with adenocarcinoma of unknown primary who received platinum doublet chemotherapy. Orienting primary sites either IHC or cDNA microarray in patients with CUPs is not good enough, we need to examine survival benefit when applying organ-oriented standard chemotherapies for patients with CUPs. Our results may encourage a prospective randomized trial to compare standard platinum doublet chemotherapy with treatment determined by the IP. This approach may assist in developing new treatment strategy compared to a single arm platinum combination trial.

## Methods

### Patients

Patients diagnosed with CUPs between 1997 and 2008 at the National Cancer Center Hospital were selected from our database. The following procedures were performed and criteria applied for diagnosing CUPs: careful physical examination by physicians, urologists, dermatologists, otolaryngologists, and gynecologists (female patients); computer tomography, mammography (female patients), gastrointestinal endoscopy, colonoscopy or stool occult blood testing if colonoscopy was not feasible; urinary cytology with negative results; biochemical and blood tests; no elevated levels of organ-specific serum tumor markers, including cancer antigen 125 (CA 125) or PSA; and histologically confirmed metastatic cancer. All the patients undertook biopsy (core needle biopsy or open biopsy) before first-line chemotherapy for diagnosis.

The eligibility criteria for this study were as follows: patients who received platinum doublet chemotherapy as first-line chemotherapy; patients diagnosed with adenocarcinoma; performance status (PS) 0–2; age ≥18; and patients who provided informed consent for their tissue samples to be used for the analysis. Exclusion criteria for this study were as follows: patients with favorable subsets [1], i.e., females with axillary lymph node metastasis; females with elevation of serum CA 125 levels with peritoneal metastasis; and males with elevation of serum PSA levels, elevation of serum alpha fetoprotein, human chronic gonadotropin, or suspected of extragonadal germ cell tumors. Male patients with intensive physical examinations, including biopsy for prostate cancer, without significant elevation of serum PSA levels and without evidence of osteogenic changes in bone were included in this analysis. Females without significant elevations of serum CA125 and with no evidence of disease in genital organs or the peritoneum were also included in this analysis. This study was approved by the institutional review board of the National Cancer Center and conducted in accordance with Japanese ethics guidelines for clinical and epidemiological studies, which took effect in August 2007. Informed consent for all the participants were done by the patients or their family before starting this research by sending mail to allow tissue samples use for clinical research.

### Immunohistochemistry and polymerase chain reaction

We used formalin-fixed paraffin-embedded tissue samples for IHC and gene analysis. For IHC, paraffin sections were treated with hydrogen peroxide to inactivate endogenous peroxidases after deparaffinization in xylene and rehydration in ethanol. Slides were placed in 10 mmol/l of citrate buffer at pH 6.0 (REAL™ Target Retrieval Solution; Dako, Tokyo, Japan), then autoclaved for antigen retrieval. Primary antibodies were incubated for 1 h and a secondary antibody was used to detect protein expression using EnVision™ (Dako). Finally, a substrate-chromogen mix was used for visualization of the immunoreaction. Meyer's sour hematoxylin was used as the counterstain. The antibodies used were as follows: CK7 (clone OV-TL 12/30, 1∶100; Dako), CK17 (clone E3, 1∶40; Dako), CK19 (clone RCK108, 1∶50; Dako), CK20 (clone KS20.8, 1∶50; Dako), ER (clone 1D5, 1∶50; Dako), PgR (clone 1A6, 1∶50; Dako), CDX2 (clone CDX 2-88, 1∶100; Abcam, Tokyo, Japan), TTF-1 (clone 8G7G3/1, 1∶100; NeoMarkers, Fremont, CA, USA), WT-1 (clone C-19, 1∶500; Santa Cruz Biotechnology, Inc., Paso Robles, CA, USA), PAX8 (clone 10336-1-AP, 1∶200; Proteintech, Chicago, IL, USA), mammaglobin (clone 304-1A5, 1∶200; Dako), GCDFP-15 (clone 23A3, 1∶50, Dako), PSA (clone 304-1A5, 50∶1; Dako), and uroplakin (clone AU1, 1∶50; Abcam).

Immunohistochemical evaluation was performed by three persons (K. H.,Y. S., and H. T.) blind to clinical information; >10% positive cancer cells was considered positive at any intensity. One pathologist (Y. S.) also evaluated whether identification of the primary tumor site based on the IP was adequate when referencing hematoxylin-eosin stain results.

Genomic DNA was extracted after microdissection at the laboratory of SRL (Hamura, Japan) or Pathology and Clinical Laboratories Divison at the National Cancer Center Hospital. K-*ras* gene (accession no. NM_033360.2) mutations at codon 12 or 13 were detected by polymerase chain reaction (PCR) and direct sequencing (SRL). Deletions of codon 746–750 in exon 19 and mutations of codon 858 in exon 21 of the *EGFR* gene (accession no. NM_005228.3) were detected by high-resolution melting analysis at our institute [41].

### Development of a panel using immunohistochemical stain results and polymerase chain reaction

We developed an algorithm using the 15 biomarkers to determine the primary site of the CUPs based on previous reports (Figure 1) [9], [10], [13], [14], [15], [16], [17], [18], [19], [20], [22], [24], [25], [27], [28], [40], [42], [43]. Using the algorithm, primary identified, surgically resected adenocarcinomas from 12 different sites of 107 samples were analyzed to calculate sensitivity and specificity of this algorithm. Tumors which did not match this algorithm except followings were classified unclassified; tumors with all markers-negative except for CK19 and ER were classified as breast profile; tumors did not match this algorithm but CDX2-positive were classified as digestive profile; and tumors with all markers-negative except for uroplakin were classified as urothelial profile.

Using the algorithm, all 71 cases of unknown primary adenocarcinomas were classified into one of the following 9 IPs: lung profile, gynecological organ profile, i.e. ovarian epithelial carcinoma, uterine body carcinoma, and cervix carcinoma, digestive profile, breast profile, prostate profile, urothelial profile, biliary tract and pancreatic profile, liver/kidney profile, and an “unclassified” profile. Initially, all cases were classified as CK7+/CK20+, CK7+/CK20−, CK7−/CK20+, or CK7−/CK20−, and subsequently the samples were further analyzed using the other 13 markers. Patients with digestive profiles were also examined through K-*ras* mutations, and patients with lung profiles were also identified through *EGFR* mutations. All new data has been deposited in GenBank (accession no. NM_033360.2 and accession no. NM_005228.3).

### Statistics

The response evaluation was retrospectively performed according to the World Health Organization criteria [44] by K.H. blinded to the IP results. Briefly, a partial response is defined as a 50% reduction in the sum of the tumor cross products. Progressive disease is defined as a 25% increase in the sum of one or more of the tumor deposits. Progression-free survival (PFS) was defined as the time from initiation of chemotherapy until detection of progression. Deaths of patients who died without evidence of a recurrence were treated as events. Patients who were lost to follow-up were treated as censored observations. The overall survival (OS) period was defined as the time from chemotherapy until the date of death or the most recent follow-up. Patients who were lost to follow-up were treated as censored cases. Median PFS and median survival time (MST) were calculated using the Kaplan-Meier method, and significance was determined using the log-rank test. For univariate and multivariate analyses, the Cox proportional regression model was used. All calculations were performed using SAS version 11.0 (SAS Institute, Inc., Cary, NC, USA).
